# Sense and the science of nineteenth-century environmental determinism

**DOI:** 10.1177/00732753251408130

**Published:** 2026-05-23

**Authors:** Brooke Penaloza-Patzak

**Affiliations:** Faculty Center for Transdisciplinary Historical and Cultural Studies, University of Vienna, Austria

**Keywords:** Science and empire, environmental history, environmental determinism, sensory studies, history of collections, knowledge regimes, museum anthropology, history of scientific practice

## Abstract

In 1865, nineteen-year-old William Healey Dall was appointed second in command of the Western Union Telegraph Expedition Scientific Corps. The expedition aim was to gauge the feasibility of running a telegraph wire overland from California to Europe by way of the Bering Strait. U.S. Congress, operating under the aegis of the Smithsonian Institution, commissioned a Scientific Corps to capitalize on the expedition infrastructure through the collection of regional things, intelligence, and beings. This contribution follows Dall’s use of the mollusks he collected as specimens into the scientific discourses they were called upon to shape. It unpacks how specimens and specimen collections were leveraged as evidence for environmentally deterministic perspectives, and how object-based classificatory practices and theories of biotic development hatched in the natural sciences were brought to bear on analyses of human culture. In so doing, it also proposes a means of excavating how the mode of subjectivity that is sensation, accumulated in the everyday work of science-making, figured alongside more formally trained methods and theories into the formation of scientific knowledge about environs beyond the ken of Euro-American science. The premise here is that the analysis of the things, practices, and theories employed in one researcher’s diagnosis of Beringia as an adverse environment, alongside the field diaries, correspondence, and other materials relating to his time in the field, has the potential to lend unique insight into how embodied experience entered the purview of what has historically constituted objective knowledge about the environment.

## Introduction

Mollusks are ubiquitous. They and their traces – fossil, food, refuse, adornment, and otherwise – inhabit a stunning range of environments, from the heights of mountain peaks and tree branches to the depths of the ocean’s midnight zone.

^1^ In the course of their lives, mollusks also become implicated in myriad relationships with their surroundings, and these relationships can continue long after the mollusk’s death. They live off plankton and synthesize proteins and calcium carbonate into intricate patterns that come down to us in their vast array of shell forms, patterns, and hues. These shells, in turn, have the potential for far-reaching afterlives, as fertilizer, a home for other organisms, or as an object of desire and research data point for human scientific collections.

The history of mollusk-based research consequently intertwines geographic spaces, geologic eras, scientific disciplines, and ways of knowing. Long before they were collected, classified, described, accessioned and registered, sketched and engraved in the name of science, the mollusks at the center of this history resided by the hundreds of thousands in a particular environment: the Bering Strait region, henceforth referred to as Beringia. In the mid-nineteenth century, this region’s natural resources became the object of mounting U.S. commercial and political interest. Bringing together methods and considerations from the history of science, environmental history, museum anthropology, sensory studies, and economic and social history, this piece relates how the mollusks of Beringia became imbricated in nineteenth-century discourses on biotic and cultural development, and were ultimately enrolled as evidence that conditions of life in this place were substandard, and as such arrested the development of its native inhabitants, other-than-human and human alike.^2^

This work is conceived as a reply to Sadiah Qureshi’s call for more a thoroughgoing “‘peopling’ of natural history” and a contribution to the historicization and critical analysis of normative knowledge regimes that scholars like Jen Rose Smith, Nicole Starosielski, and Hi’ilei Hobart are carving out at the intersections of environmental history, Indigenous and cultural studies, and geography.^3^ The aim is to begin unpacking how and why things, specimens, were leveraged as evidence for environmentally deterministic perspectives in the nineteenth-century life sciences, particularly in light of the coincident emergence of what Lorraine Daston refers to as the “moral economy” of scientific objectivity.^4^

The contribution takes up Steven Shapin’s recommendation that those desirous of insight into how objectivity fits into the formation of scientific knowledge shift their sights to the phenomena of intersubjectivity, or how “free and practical interactional assent about what is” comes about. He suggests we do so by embarking upon more systematic analysis of how specific modes of subjectivity figure into “concrete knowledge-making practices” in the “everyday life of science-making.”^5^ The premise here is that analysis of the things, practices, and theories employed in one researcher’s diagnosis of Beringia as an adverse environment alongside the field diaries, correspondence, and other materials relating to his time in the field has the potential to lend unique insight into how embodied experience entered the purview of what has historically constituted objective knowledge about the environment.

In keeping with the aim of this special issue, this contribution follows mollusks into the environmentally deterministic discourses they were called upon to shape. The first section delves into nineteenth-century Beringia as an environment as well as object of imperial, economic, and scientific interests.

The following section unpacks one naturalist’s taxonomy of mollusks as founded on the assumption that Beringian environs were adverse and had stunted biotic development. The next section takes a closer look at this assumption in conversation with that naturalist’s diaries and other unpublished and published expedition-related material to exhume his embodied experience of making science in the field. The penultimate section brings these two strands of inquiry into conversation with roughly parallel investigations the same naturalist carried out using mollusk remains to reconstruct the history of human cultural development in Beringia.

The naturalist in question is William Healey Dall (1845–1927), a malacologist memorialized in, among others, the patronyms for the Dall Sheep and Dall’s Porpoise. Dall was also active in the institutionalization of U.S. anthropology, yet, as geographer Peter Martin has pointed out, his scope of influence in that field remains little understood.^6^ This contribution take Dall’s Strait-based research as a point of entry for thinking about longer-term patterns in the use of objects as evidence in the sciences, foregoing neat disciplinary arcs in favor of engaging with ambiguity, in this case the complicated means by which methods for “reading” mollusks as data informed a range of knowledge-making projects. Attending to not only the scientific and material, but also social, economic, and political circumstances that facilitated Dall’s investigations in Beringia, this research demonstrates that he ultimately took on a key role in shaping scientific perceptions about that region and the living and once living, other-than-human and human beings who inhabited it.

## Science and empire on the Strait

As a rule, maritime straits link larger bodies of water while bounding lands on either side, thereby mediating between countless human and more-than-human worlds. The former confer many kinds of significance upon straits: environmental, cultural, economic, and political to name just a few. Straits also harbor unique research opportunities: occasion to study the interplay of organic life and natural phenomena, past and present movement and isolation in and on water, littoral, and land.^7^ The history of practitioners who set out with a special interest in the invertebrates of intimately bound maritime environments is long. Prominent nineteenth-century exponents include Charles Darwin and Karl Möbius, and the best-known of their labors is perhaps the latter’s investigations on Kiel Fjord that gave rise to the concept of biocenosis, and helped usher in a turning point in late nineteenth-century biological thought.

Rich in maritime resources and far easier to commandeer than oceans, human powers have long vied over straits, and Arctic straits remain heavily contested to date.^8^ The Bering Strait, a mere fifty-five miles at its narrowest, spans the continents and histories of Asia and North America, and first excited the European colonial imagination between the late 1720s and 1740s when scientific expeditions, deployed to find the easternmost border of the Russian crown lands, encountered first the strait, then the land beyond.

**Figure 1.**
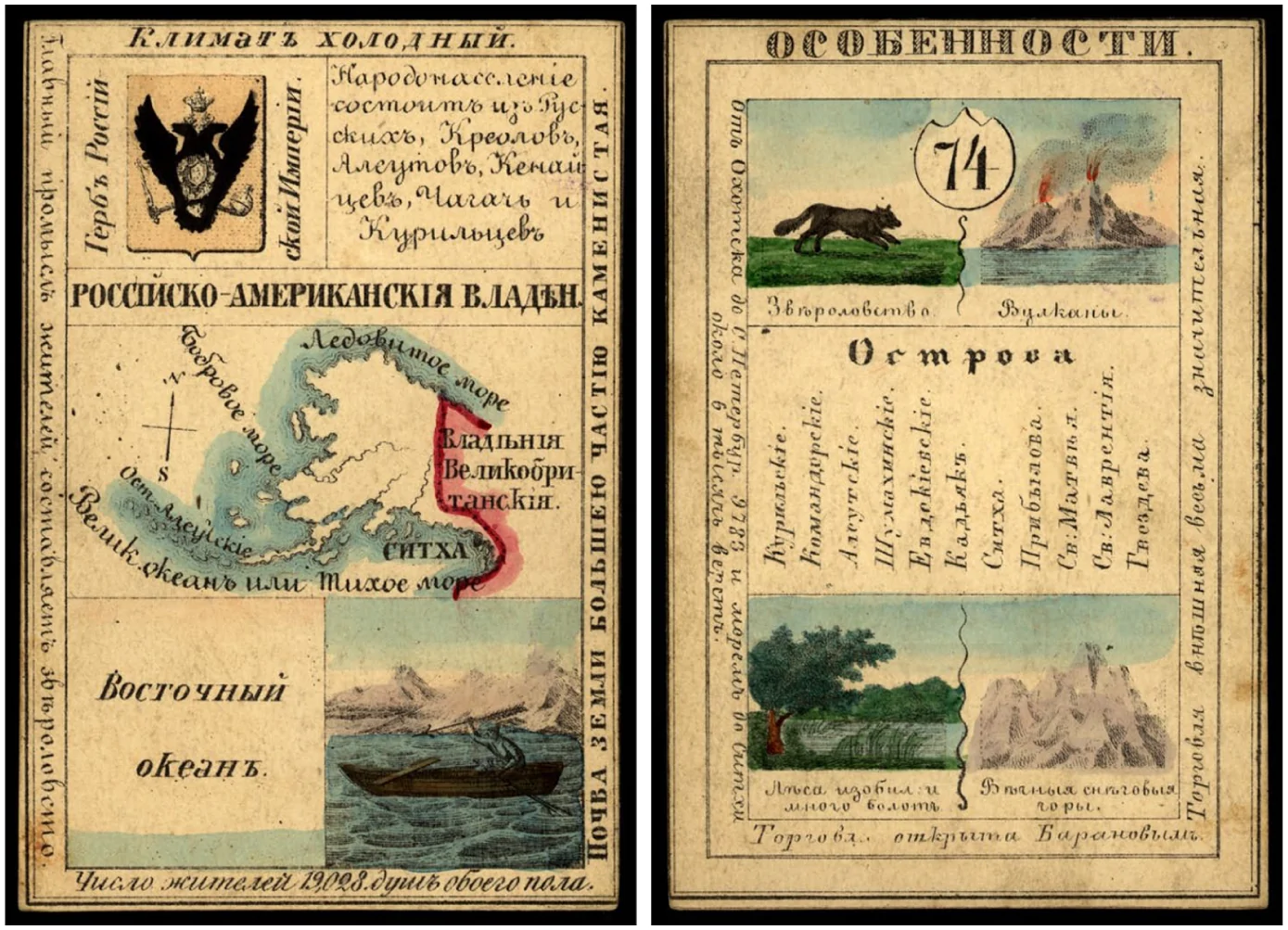
“American Territory,” front and reverse of card advertising Russian America’s attributes and natural resources on the eve of its sale, 1856. Library of Congress World Digital Library Collection, LCCN 2018689230.

The land beyond abounded with volcanic fields, forests, marshlands, seas, mountains, and all manner of plants and animals, a site of age-old movement and the domain of Indigenous groups with well-established trade relations, both with one another and with the British colonies to the east. Commercial interests entwined and outpaced the Russian crown, capitalizing on the land and its inhabitants, while government involvement in regional affairs increased in response to the number of subjects engaged there.^9^ In 1799 the Russian Empire declared the land its own: Russian America, to be administered by the Russian-American Company (RAC) (Figure 1). By the 1850s, however, the trapping industry had begun to falter; overhunting was decimating the sea otter population, seals too were dwindling, and RAC subsidies plagued the Russian treasury.^10^ U.S. expansionary interests were nonetheless piqued. The young republic had just come up against its westernmost limit in the form of the Pacific Ocean, and envisioned Russian America a potential trove of unplumbed resources.^11^ Dall’s work in Russian America, the Bering Strait, and eastward into Russia offers a glimpse into the potential for scientific research undertaken under the cover of expeditions awakened by commercial or political interests to help legitimate the expansion of the same.

Dall left high school in 1862 to work as an office boy on Boston’s now defunct India Wharf, thereby earning the means to train as a special student with Jeffries Wyman and Louis Agassiz at Harvard University. The latter was Professor of Zoology and Geology. His Museum of Comparative Zoology was a “fortress against evolution,” and the first address in the United States for an education in zoological taxonomy.^12^ Agassiz’s students learned speciation was the result of progressive creations. Their task was to decipher the intelligent design that was “built into every living thing,” and the act of classification was tantamount to “the literal interpretation” of the plan of creation.^13^ Wyman was Professor of Anatomy and Physiology. A belief in natural rather than divine selection was quickly gaining ground in his museum and workshop for comparative anatomy, and students were trained to seek the relationship between adaptive morphological differentiation and evolution as such.^14^

Two years later Dall, now assistant at the Illinois Central Railroad Land Office and moonlighting as a cataloger at the Chicago Academy of Sciences (CAS), lamented his scientific prospects.^15^ If he “only had the chances” afforded some contemporaries he could “be something, or somebody.”^16^

That chance soon arrived thanks to mentor and CAS Director Robert Kennicott. Western Union’s second attempt to run a telegraph cable through the Atlantic had snapped, and they were now organizing the Russian American Telegraph Expedition (i.e., WUTE: the Western Union Telegraph Expedition) to determine the viability of an overland cable run the other way around, across the Bering Strait.^17^

U.S. Congress, operating under the aegis of the Smithsonian Institution, capitalized on this opportunity to engage Kennicott as head of the Scientific Corps comissioned to make use of expedition infrastructure to conduct exhaustive regional land surveys and collect regional intelligence, fauna, flora.^18^

Dall, nineteen years old, was skeptical about the undertaking, but with few employment prospects and full of confidence in both Kennicott and the manifest destiny of U.S. science, he enlisted as lieutenant naturalist with the Corps in March of 1865.^19^ Their assignment was to survey the geography, geology, and natural resources, including plant and animal life, in and around Russian America and the Bering Sea. The commission also afforded Dall the opportunity to pursue his life’s passion en route, at ports of call and in the field: collecting, drawing, describing, and classifying mollusks.

He commemorated their departure with a riff on a popular poem: “‘Westward the course of Empire takes its way,’ and Science follows with her starlit train. Stern mistress well beloved, whose generous hand Deals . . . Such treasures vast I may not idly refute to grasp.”^20^ En route, Kennicott appointed Dall Chief of Explorations in Russian America. His investigations, which ventured into ethnography and archaeology and beyond the Strait to Kamchatka, Russia, plumbed the bounds of natural history and Russian America alike, and the collections he made during this and subsequent expeditions formed the basis for his earliest work on biogeographic distribution and organic development.

## Beringia’s true limpets: anatomy as evidence of adverse environs

**Figure 2.**
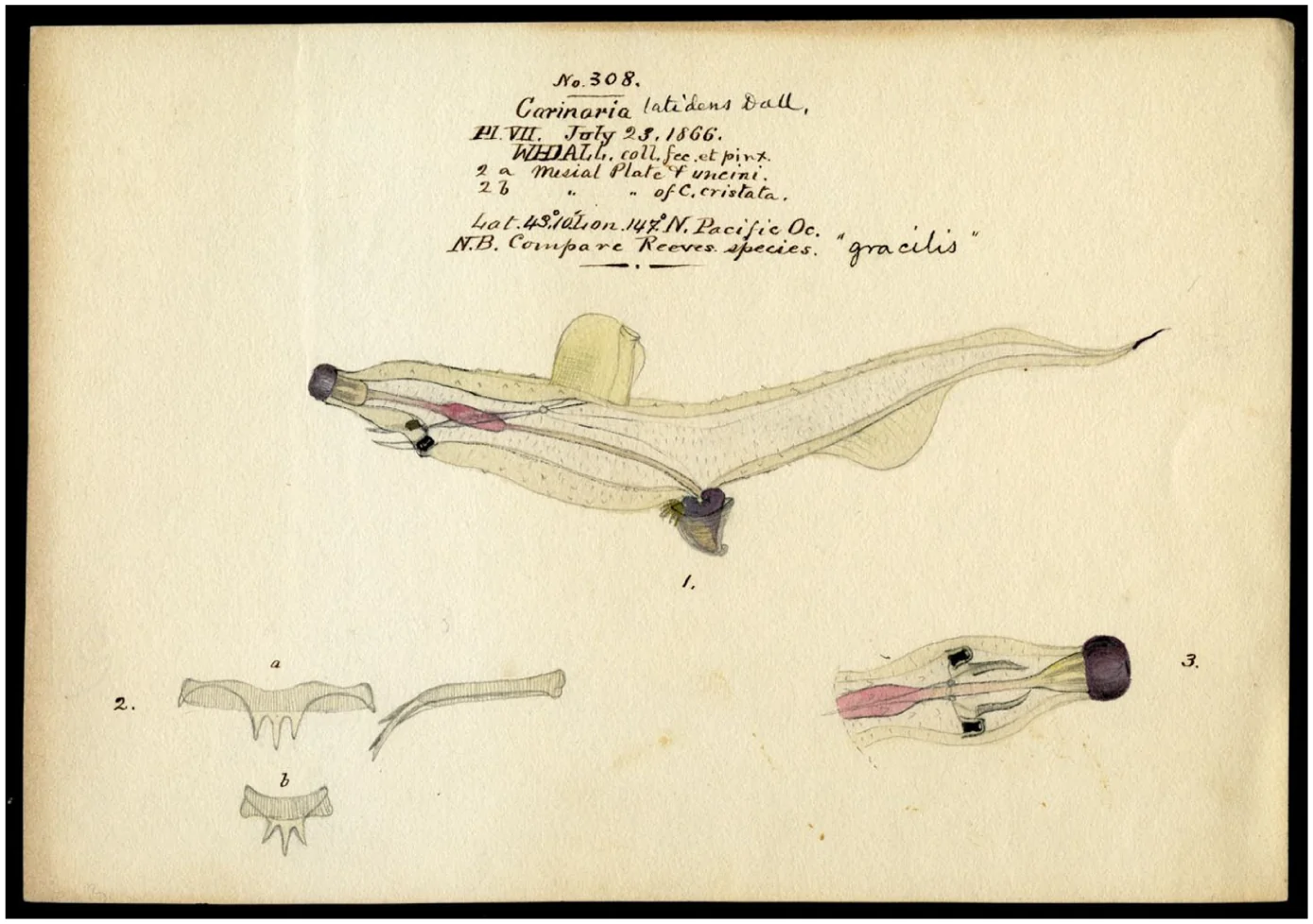
Dall’s diagram of a “fine though small Carinaria,” now *Carinaria cristata*, or “glassy nautilus.” The creature’s body is translucent, and the shell is the cone-like structure just to the right of the number 1. Sketched by a “sick and miserable” Dall en route to Russian America, July 23, 1866. Image courtesy of Smithsonian Institution Archives, Image no. SIA2012-7918.u.

Few naturalists had studied the mollusks of the North Pacific and Arctic in situ, making this a valuable opportunity for a young researcher eager to establish a reputation.^21^ Dall’s diaries, field notes, and detailed color diagrams document a fascination with mollusks in their entirety, yet mollusk shells, which are far easier to clean, dry, prepare, preserve, and transport than their fleshy insides, are all that remain today of the corporeal basis of his analyses. As one naturalist remarked, one could not simply “wrap a moist and mucose animal in note-paper and expect it to reach its destination like an invitation for dinner.”^22^ A case in point was the glassy nautilus they captured in an overnight trawl some one thousand miles off the coast of Oregon. Given the right conditions, a soft bodied creature such as this could survive one, maybe even two days onboard, but died and withered soon thereafter, leaving as evidence of its existence only its shell and whatever descriptions had been put to paper in the meantime (Figure 2).

Every mollusk’s shell is the unique work of its mantle, and it is this, together with the mollusk’s more ephemeral fleshy parts, that constitute the basis of scientific classification. Contemporary taxonomy relies on a combination of genetic, evolutionary, and visual analysis, but mid-nineteenth-century classification depended on looking alone. Dall’s studies at Harvard had afforded ample instruction in the intimate analysis of organisms’ characteristics, the relations between them, and how to transform these impressions into classifications (on objects and classification see also Raphael Uchôa, and Kuang-Chi Hung and Leo Chu in this issue). Agassiz regarded the training of naturalists as comprised “chiefly [of] learning how to compare,” and was known to initiate students into the art of classification and relying above all on their eyes by way of collections-based exercises. For some it was trial by fire: sorting mixed lots of hundreds of shells; others he might seat at a table with a single specimen for days at a time, stopping by at intervals to hear what more had been observed, until he was satisfied the pupil had acquired competence in seeing. This would be followed with another specimen from the same group, and then another, until the student had succeeded in describing an entire family, and in so doing absorbing both how to differentiate between representative and merely incidental characteristics, and Agassiz’s developmental philosophy.^23^

In practical terms, morphological classification hinges on access to specimens, precise visual apperception of anatomical and physiological detail, and the ability to identify patterns in and between specimens. The determination of taxic relationships entails an added speculative component, laying bare the taxonomist’s theoretical framework. Dall’s work on the aquatic snails commonly known as true limpets, or what he classified as *Docoglossa*, is a fitting case in point. He began with extracting live mollusks and shells of the deceased from the sea. The former he attempted to preserve in kegs or tins of alcohol, soldered shut and stored in wooden encasements. When preservation failed, he boiled the creatures to glean the shells from their bodies. Dried shells he stowed in hand-rolled paper tubes, and wet and dry material alike were shipped eastward.^24^ At the end of his travels, Dall followed his collections to the Smithsonian, where he worked up the material as a resident of the castle towers. “Working up” refers the process of visually analyzing, describing, cataloging, and accessioning things into museum collections. These activities form the foundation of specimen-based knowledge, and as such are a fitting juncture at which to seek the empirical foundations upon which Dall based his analyses of Beringia, which he reckoned “undiscovered country where naturalists are kings.”^25^

Dall’s natural history of true limpets hinged on two interdependent premises: first, that the order descended from a single point of origin; and second, that the larger and more anatomically complex the species, the more advanced its position in the order’s developmental history.^26^ Dall judged the *Docoglossa* of Beringia and the North Pacific comparatively simple and small in scale, and interpreted this as indications that this was the order’s geographic point of origin. He further theorized that the order had spread southward from there throughout the Pacific, with species developing in size and complexity with the encounter of increasingly “favorable” environmental circumstances.^27^ Noteworthy with respect to Dall’s conception of the mollusk as an organism and his use of mollusks as evidence is that he considered the mollusk’s shell and anatomy as mutually informed, obviating the need to preserve their fragile bodies and rendering developmental relations discernable from shells alone.^28^

Malacologist David R. Lindberg has interpreted Dall’s early work on *Docoglossa* as indication that he was largely unfamiliar with the literature on evolution at this point in his career, and ascribes Dall’s evolutionary philosophy in large part to Agassiz.^29^ Newer scholarship suggests he was more interested in evolutionary theory than previously recognized, and far more influenced by William Stimpson, a staunch but discrete Darwinist likewise affiliated with the CAS, who had worked as naturalist in Beringia as part of the United States North Pacific Exploring Expedition of 1853–6 (on this likewise Smithsonian-affiliated expedition see Hung and Chu’s contribution in this issue). The two met to discuss the subject shortly before Dall’s departure, and also maintained steady correspondence throughout the expedition.^30^ As Dall later put it, Stimpson “stood ready to welcome the theory of evolution with all the light it shed in dark places,” and was something of a “scientific missionary, a biological bishop, *in partibus infidelium* [in the lands of unbelievers].”^31^

Thinking beyond malacology and zoology to moreover consider Dall’s time with Wyman in the workshop for comparative anatomy, we gain a broader perspective on his early practice and logic as a product of simultaneous training with other-than-human and human specimens. Likewise significant is that though Agassiz and Wyman differed in how they explained the diversity of life, both considered the complexity of structure the key to divining natural history. To quote the latter, evolution most likely proceeded by “progressive series of differentiations” in which “simple forms were not only the precursors, but the progenitors of the complex ones.”^32^

**Figure 3.**
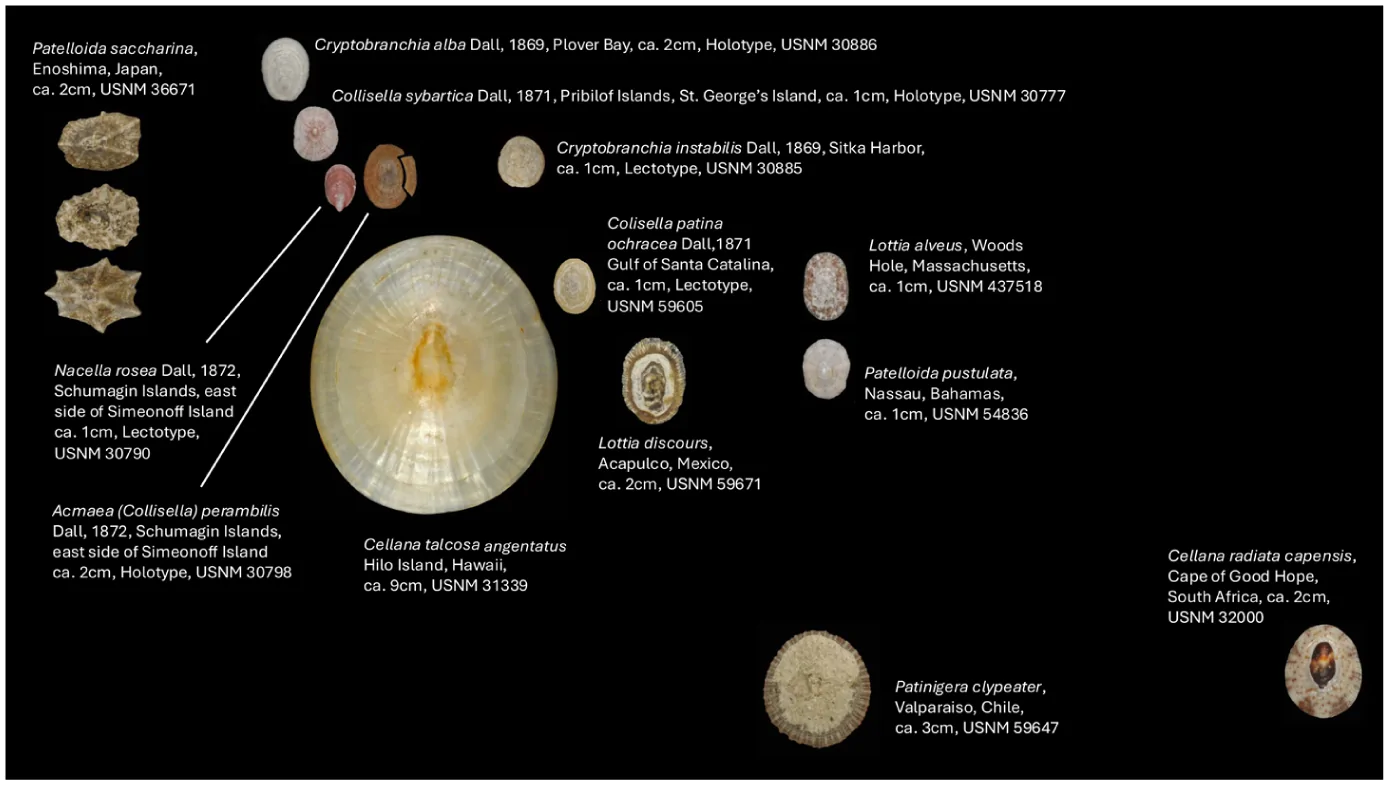
Representative selection of scaled and geographically plotted “*Docoglossa*” assembled by Dall, demonstrating no conclusive evidence that Beringia’s mollusks were smaller or less complex than those from other regions (species authorship and year of description as given in the National Museum of Natural History [NMNH] database, place names as historically noted). Author’s composite created from images courtesy of NMNH Department of Invertebrate Zoology.

Lindberg has since reclassified *Docoglossa* as *Patellogastropoda* and urges contemporary malacologists to “carefully consider” before adopting Dall’s taxonomies. As the penultimate section demonstrates, caution should likewise be exercised with regard to the taxonomies Dall theorized beyond the field of malacology. Dall’s progressive evolutionary philosophy had “tremendous implications” for classifications.^33^ The Smithsonian’s National Museum of Natural History now houses more than 400 *Patellogastropoda* lots that Dall procured in the course of his career. Given his equation of mollusk shells and bodies, a representative survey of those he classified as *Docoglossa* arranged according to geographic origin offers intimation of the predicament: the comparative size and complexity of these shells – many among them holotypes, the very organisms upon which he based new species descriptions – belies the claim that they were as a rule smaller or less complex than *Docoglossa* elsewhere, therewith confounding the rationale of a southward developmental trajectory (Figure 3).^34^

To review, this section has established that Dall did indeed take an interest in developmental discourses of the day and believed that the key to deciphering the historical development of, and relationships between, organisms lay in distinguishing a progression from simple to complex forms. He established and refined this approach over the roughly two-and-a-half years of collections-based training that preceded his enlistment with the Corps, first as a special student with Wyman and Agassiz, then as a cataloger at the CAS. This offers crucial insight into the practical and theoretical tenets that shaped his conception of how the natural world functioned. It does not, however, bring us any closer to accounting for the premise underlying his natural history of the *Docoglossa*, namely that Beringian environs stunted the development of endemic orders.

At the time, the concept of environment was still coming into being. Karl Möbius, Heinrich Adolf Meyer, and others were just beginning systematic investigations into the relationship between marine beings and the waters they inhabited. There was little empirical evidence of how organisms were affected by their surroundings in general, much less the physical and chemical factors that comprised specific environments.^35^ How then to interpret Dall’s knowledge that Beringia had inhibited Docoglossan development?

The section that follows proposes that Dall’s knowledge was based in part on subjective experience, and, as per Shapin’s suggestion, embarks upon a systematic analysis of how subjectivity figured in Dall’s “everyday life of science-making.”^36^ Shapin’s analyses have centered on the mode of subjectivity that is taste, harnessed for instance by the Wine Aroma Wheel, a “homespun intersubjectivity engine” with which wine connoisseurs the world over have come “reliably to assign stable descriptors to wine odors and tastes.”^37^ Delving into the negotiations of Beringian climate and environs amply chronicled in Dall’s diary, analysis here centers on the mode of subjectivity that is physical sensation. The objective basis for Dall’s inferences about Beringia’s effect on development is neither so collectively nor materially manifest as that of the Aroma Wheel. Indeed, it is implied, and it is precisely the ill-defined root of this certainty, in conjunction with Dall’s subsequent influence within nineteenth-century science, that renders a more thoroughgoing understanding of its authority so desirable.

## Beringian environs, beyond the ken of nineteenth-century sciences

“We heed not the cold nor the Arctic icy wall,” so begins a ditty on the final pages of the diary in which Dall chronicled his first months in Beringia.^38^ Truth be told, however, this diary and those that followed recount that the day-to-day endeavor to navigate work and life in Beringia was far more daunting than this ode to the Scientific Corps was wont to betray. He did indeed heed the cold, so much so that the “clasp” of these unfamiliar environs steeped into his conception of Beringia’s impact on organic life more generally.

We have established several of the more intentionally “trained” ideas and practices that informed Dall’s outlook on Beringian environs, as well as some of the cited as evidence for that knowledge.^39^ This section employs Dall’s diaries and other expedition-related materials to establish that the experience of science-making in this new environment was also instrumental in shaping his sense of what constituted objective knowledge about this place.

Between 1865 and 1868 Dall spent more than two years in Beringia. His published expedition report conveys a verve in keeping with the aforementioned ode to the Corps,the long entagled history of imperial science, and capitalist extraction at the poles, and “impulse to test and evidence masculine mettle against the elements.”^40^ He spurned, for instance, the wont of “pioneering spirit,” among U.S. denizens who derided Alaska’s economic potential merely because it was known as “a ‘cold’ country.” “The best plan in cold weather,” was to simply “face the wind boldly,” and snow itself was the best remedy for frostbite, used to slough off affected skin it left a “brown stain resembling sunburn, and quite as ephemeral.” ^41^

In private writings, however, it is evident that Beringia engendered within him a worrisome sense of impotence and unease.^42^ It is not that Dall was a stranger to cold, ice, or snow. He spent the winter before the expedition in a cheap Chicago boarding house with grudgingly rationed coal, and deemed seven degrees Fahrenheit “still warm.” But clouds, rain, and fog that were heavier, and gales far stronger than those to which he was unaccustomed likewise fell within his notion of adverse weather, and rendered the everyday life of science-making in this new place a perpetual struggle.^43^

At the time, standardized weather record-keeping and forecasting were still in their infancy.^44^ Knowledge about weather conditions and patterns in any given place was the province of inhabitants, and perhaps longer-term visitors. The same applied to how to navigate those conditions. Dall was a fervent upholder of objective science. He was attentive to the difference between facts based on the observation of live organisms in the field, those based on specimen-based research in the museum, and those adopted from others, and ever wary of theory and opinion.^45^ In an era anxious to establish the pertinacity of scientific truth, care in such matters was an asset Dall diligently cultivated, and this was Kennicott’s motive in choosing him as second in command.^46^ Still, the charge to apprehend and interpret the life and environs in this place so beyond the ken of everyday Euro-American science was rife with invitation for subjectivity, and Dall’s diaries afford entry into this aspect of his scientific practice.

On a steamer three days south off the Chukchi Peninsula in late September of 1865 Dall noticed the mercury in the barometer he had recently learned to calibrate had begun to fall. Fellow members of the Scientific Corps had already disembarked at their posts, and he and expedition artist Frederick Whymper were now steaming northeast in the company of Captain C. M. Scammon’s Marine League division. He set to making a cap from an old felt hat, scrap flannel, and a recently trapped muskrat.^47^ By the next day it was evident that his wool mittens were likewise inadequate: these he lined with an Arctic fox memorialized as “the meanest fur [he] ever saw.”^48^

They reached Plover Bay on September 25, “beating in against an icy wind” blowing “out of the very throat of . . . Norton Sound,” and made the acquaintance of area residents two days later when “Nocum, Eskimo [came] aboard with . . . his tribe.” Nocum’s given name was Norman Walker, and “Eskimo” a conflation of the Yupik and Inuit to whom the bay was home.^49^ The group had come, among other things, to trade, and Walker and Dall struck up an acquaintance, the former guiding and interpreting the latter’s information and object-gathering efforts, until the company steamed onward to Kamchatka. Dall and company decamped one month later to wait out winter in San Francisco.^50^

Plans to return, this time remaining through winter, began anew in June of 1866.^51^ Dall set to ordering a microscope, shirts, ammunition, medical supplies, and alcohol for wet specimens and to trade. He went to the tailor and reviewed and re-reviewed the contents of his scientific chests. This time he also intended to record meteorological data, and to that end traveled with thermometers and not one but two barometers.^52^

Mid-August found the outfit once again in Plover Bay, surveying and collecting with the aid of Walker. The weather began to turn a few weeks later and Dall, already vexed by cold and wet, had destroyed his only boots surveying a craggy mountain peak; his feet were turning “a little blue.” The outfit bequeathed one of their stoves to Walker as payment in kind and set sail for Russian America and an eagerly anticipated reunion with Kennicott at WUTE headquarters.^53^ Just off the coast of St. Michael’s, however, they were met with dreadful news: Kennicott had died that past spring. There was nothing they could do other than escort his coffin aboard before disembarking.^54^

Kennicott’s death struck a “heavy blow.” Dall had no desire to remain “in this dreary country, already white with snow,” but he “loved [Kennicott] better than anyone else in the world” and felt compelled to complete his mentor’s unfinished Yukon survey.^55^ This project, undertaken in the company of Whymper and four men noted only as “Indians,” extended over another two years, bookended by winters on either side. With most of the original scientific party now scattered across British Columbia and the Northwestern Territory, he and company lodged at Fort St. Michaels, where new high winter boots, a parka, and wild reindeer sleeping bag rendered life “dull but comfortable” while awaiting wind to sail to Unalakleet.^56^

Once in Unalakleet, home to an ancient Native village as well as the RAC’s northernmost trading post, life became more challenging. The ground had been frozen solid since early October, permitting no chance of building winter quarters. Those “terrified” of succumbing to the cold took cockroach-ridden bunks in the fort, while he, Whymper, and some others pitched tents nearby or slept in their Indigenous hosts’ *Qasgiq* (men’s house) in the village; all kept watch to see whether a tin dipper of water, frozen through the night they arrived, revealed any indication of thaw.^57^ Even so, this last colonial outpost en route to the interior was not without pretense of familiar comforts: warm meals in the fort’s bakehouse, for instance, ready access to food and medical provisions, and occasional hot steam baths.^58^

Complications arising from Dall’s inexperience and lack of adequate provisions arose the moment they attempted to strike out on their own outdoors.^59^ He underestimated the number of dogs necessary to make up a sledding team, elected to steal “all [they] could lay hands on” from the village to make up the difference, underfed the animals en route to conserve rations, and he and colleagues were left to push the 600-lb scientific kit themselves when the dogs gnawed through their harnesses and returned home.^60^

Dall had intended to continue their survey and collection work throughout the winter, and their lack of progress was a source of near-constant dissatisfaction that he inevitably attributed to weather-related circumstances rather than the challenge of acclimating to work in unfamiliar circumstances. With a feeling that their work was all but incapacitated by mid-November, weeks before winter customarily set in, he took to recording meteorological observations.^61^ His life up to then had afforded no sense of how to tread on smooth ice, and the price of snowshoes rose each day he continued bargaining. In what daylight there was, his eyes were overwhelmed by the glare of the sun on ice, and snow in fall and winter, and mist in spring, and his dealings with Indigenous interlocuters had not awoken the amity that may otherwise have prompted introduction to the eyewear – of carved horn, wood, or bone with long, thin slits carved through which to see – that Arctic peoples had developed centuries earlier for this reason.^62^

In Spring of 1867 Dall received word that a Corps comrade who was still in the field had gone mad, and noted to himself that this place was “the devil for nervous minds.”^63^ The summer months noticeably eased his sense of idleness and discontent, and by the time winter set in once again they had amassed more than 1350 lots, thousands of animals and plants, many unknown to the scientific community.^64^ Dall parted ways with Whymper in mid-December, following two guides inland along “the still and frozen Yukon [river], sitting, thinking, dull and lonely, sad or silent all the day.” Barometer now broken and useless, he mused that thoughts of life on the East Coast were like “golden visions; but they vanish. Ice and snow and gloom appearing and the shadows and the silence, seem more lonely than before.”^65^

Dall ultimately reached Fort Yukon in early summer 1868, and by the year’s end was at the Smithsonian, where he commenced drafting his expedition report and working up the materials collected in Beringia.^66^ That report, much like his work on *Docoglossa*, presents the Beringian climate as an impediment to be overcome, and lends his critical insight into the foundations for this assessment. This was a physical and psychological knowledge, rooted in his struggle and anxiety to navigate day-to-day life, much less the business of science, in deeply unfamiliar environs and absent heartening norms: church, baths, a barber.^67^

Dall’s diaries help exhume the real trouble with his taxonomies, providing vital and intimate insight into how he perceived Beringian environs, and as a tool for situating that subjective knowledge alongside the practical and theoretical aspects of his practice. The diaries also intimate a problem of gargantuan proportions for the history of science: his analyses emerge as founded on something like the phenomenon that Smith has recently termed “temperate-normativity,” as a result of which Dall, unaccustomed to Beringian climes, conflated his challenges with the impact of that environment on organic life and development more generally.^68^

Dall was by no means the first naturalist to claim an empirically based deterministic correlation between the environment and history of development, nor to suggest that environments beyond the U.S. and European norms had the potential to exercise adverse effects on organic development.^69^ His malacological investigations, however, constituted some of the first field-based research into life in the Arctic and Subarctic, and therewith, a significant point of reference for those who followed. This also extends to investigations into the human history of Beringia. The section that follows unpacks the implications of Dall’s environmental appraisal in light of his work on cultural development, which likewise enrolled mollusks as evidence.

## Beringia’s shell mounds: “rejectamenta” as chronicle of environmentally inhibited culture

By the time Dall arrived on the strait, mollusks had long figured in local quotidian and sacred practices and cosmologies. They were a central component of coastal lives, diets, and economies. The people he referred to as the “Nuklukahyét,” “Tenán Kutchin,” and “Han Kutchin” wore dentalium through their septa,^70^ the Russian traders at Fort Yukon plastered their homes with the chalky white of shell-marl,^71^ and the Haida of Queen Charlotte inlaid their pipes, jewelry, masks, and headpieces, flatware, and vessels with the nacreous shell lining known as mother-of-pearl.^72^ Vestiges of these and other activities also remained along the Beringian coasts in the form of shell heaps. Drawing Dall’s work on these sites into the conversation, this section demonstrates how and why environmental appraisal borne of exploratory science in the service of state and commercial interests had the potential to bind and enmesh other-than-human and human, biological, and cultural research and analysis.

In 1860 the Smithsonian published an English translation of Adolph Morlot’s *Études géologico-archéologiques en Danemark et en Suisse*, therewith initiating U.S. scholars into the past decade and a half of European developments in empirical methods for dating the material remains of bygone generations, and sparking a flurry of interest in shell heaps, mounds, and middens as a source for insight into life in the Americas before the advent of the written word.^73^ Shell heaps could help researchers resolve long-standing questions about the origin, antiquity, and development of the first Americans, and clarify the relation of Indigenous Americans and Europeans past and present.^74^ Wyman commenced his first shell heap investigations that same year, and throughout the 1860s he and Agassiz – separately, together, and in the company of students – examined several such sites along the Atlantic where past communities had collected, worked, traded, and discarded mollusks.^75^

Over the next decade Wyman helped promote the investigation of U.S. shell heaps, insisting they were indeed anthropogenic, could be disguised by superabundant vegetation, and were indicative of bygone, indeed pre-European, waterway, settlement, and migration patterns.^76^ In keeping with Christian Jürgensen Thomsen, he maintained that strata sequence correlated with the chronological passage of time – the deepest being the oldest, and the shallowest being the most recent – and that the organic remains and artifacts within thus told a teleological story of chrono-morphological progression that blurred the lines between biotic and cultural development.^77^

**Figure 4.**
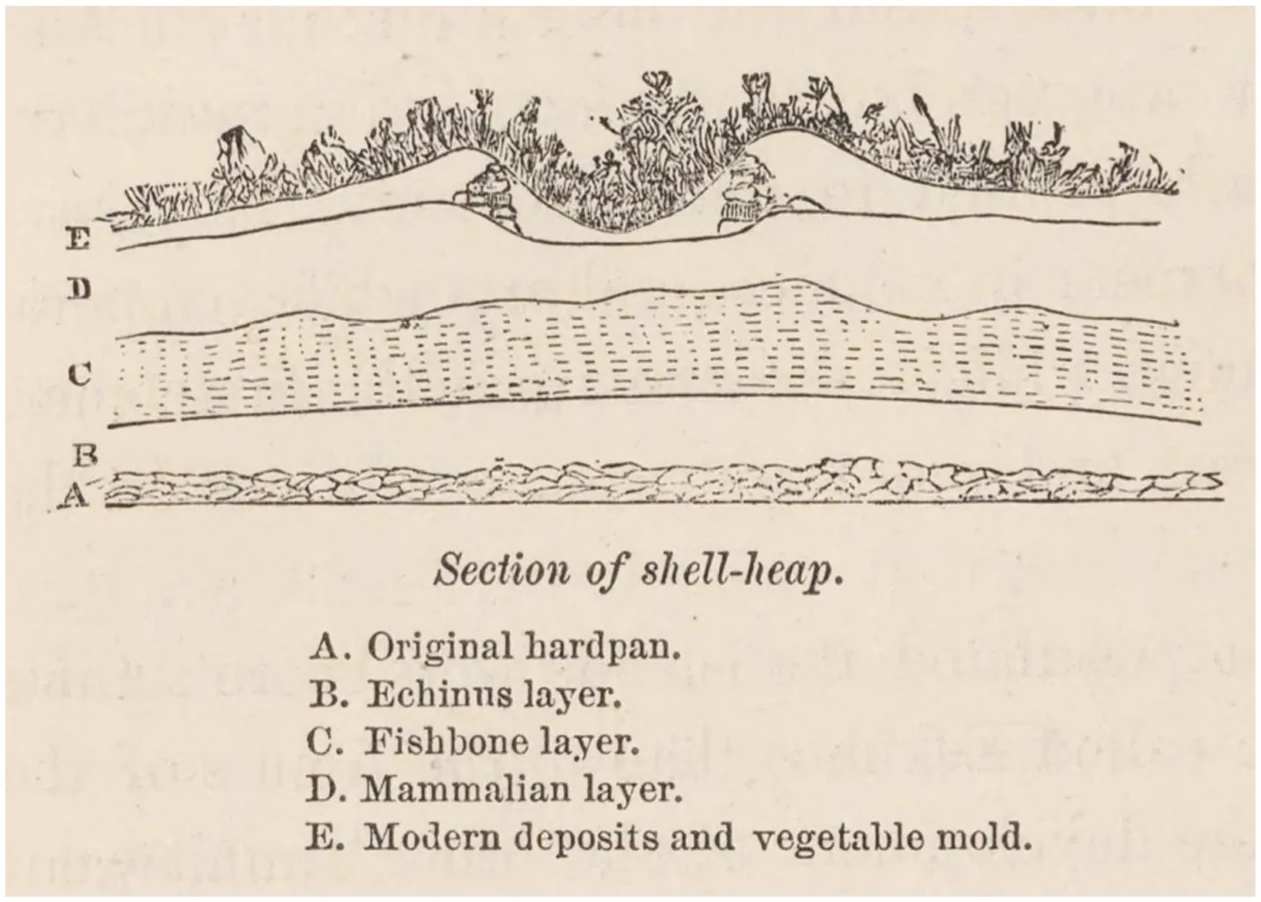
“Section of shell-heap” illustrated in Dall’s *Tribes of the Extreme Northwest*, 1877, p. 50. Image courtesy of Biodiversity Heritage Library.

Dall joined these conversations upon his 1870 return to Beringia under the auspices of the U.S. Coast Survey after noticing “incomparably rich” mounds of soil covered in “brilliant green . . . herbage” scattered all along the Aleutian Islands (Figure 4, E). Beneath he discoverd, as Wyman had suggested, layer upon layer of “bones, shells and all varieties of rejectamenta.” He soon established that these were clusters of ancient homes that had been gradually surrounded by the debris of everyday life and ultimately abandoned — material chronicles of human culture, movement and, as he would soon argue, development. From then on, whenever inclement weather prevented survey work, he and colleagues set out with picks and shovels, in storm-proof rubber coats, boots, and sou’westers, to excavate.^78^

Dall’s work with these sites took him into the very heart of debates on the origins and development of Indigenous Americans, and offers crucial insight into how object-based classificatory practices and developmental theories hatched in the natural sciences were introduced into analyses of human culture. To begin, Dall regarded the “aborigines of North America . . . [as] naturally divided into two great groups,” the “Orárians,” by which he referred to the “Innuit, Aleutians, and Asiatic Eskimo” on the one hand, and all others “universally known . . . [as] Indians” on the other.^79^ The former were “more intelligent, and superior in every essential respect” to the latter, who presumably hailed from a southwesterly clime.^80^ But where had the Orárians originated? And how and why had the three cultures differentiated?

Excavations evinced that the deepest human shell heap stratum was inevitably chiefly composed of sea urchin spines. Dall named this the Echinus Layer, equivalent to the Littoral Period, and produced by the area’s first humans (Figure 4, B). Based on the contents of this layer, he surmised that they had largely subsisted on mollusks, and migrated from one costal site to another as the supply dwindled or was succeeded by other easily attained food sources. Observing no evidence of charcoal, weapons, or seafaring vessels, he classified Littoral Period people as “similar to the lowest grades of Innuit,” without fire, nearly naked, and likely cannibals “sunk in the lowest depths of barbarism.”^81^

The second deepest stratum, appointed the Fishbone Layer, evinced an “extraordinary change in the whole character of the deposit” (Figure 4, C). The sea urchin spines were supplanted by a diversity of artifacts: traces of large-scale fishing nets, finely articulated sinkers, knives, and darts and lanceheads from the ivory and bones of sea creatures.^82^ He surmised these durable yet workable materials had stimulated “the aboriginal mind much as . . . the printing press and telegraph . . . affected modern races,” motivating an almost inexplicable leap toward civilization. Though still absent evidence of charcoal and seafaring vessels, he ascribed the makers of this layer clothing, canoes, permanent settlements, store-houses, and fires, not to mention vanquishment of the alleged cannibalism. They were harbingers of the final phase of “prehistoric” development: the Hunting Period (Figure 4, D).^83^

But how to account for such a radical leap toward civilization, so out of keeping with the plodding pace he knew from biotic development? The shell heaps themselves were stationary, precluding a move of environment. Likewise improbable was a positive influence from neighboring “Indians,” judged far too inferior to stimulate such transformation. Dall surmised, in short, that after crossing into North America by way of the Bering Strait the “Original Innuit [*sic!*]” fanned out in groups, some continuing eastward into North America, others down into the Aleutian Islands.^84^ Under the influence of “genial” environmental circumstances affording ample “opportunities for development in manners and arts,” the former, now “Continental Innuit,” flourished. The latter, isolated on the Aleutian Islands in a “ceaseless struggle” to wrest sustenance from the unrelenting environment, stagnated; these he classified the “Low Innuit.”^85^

**Figure 5.**
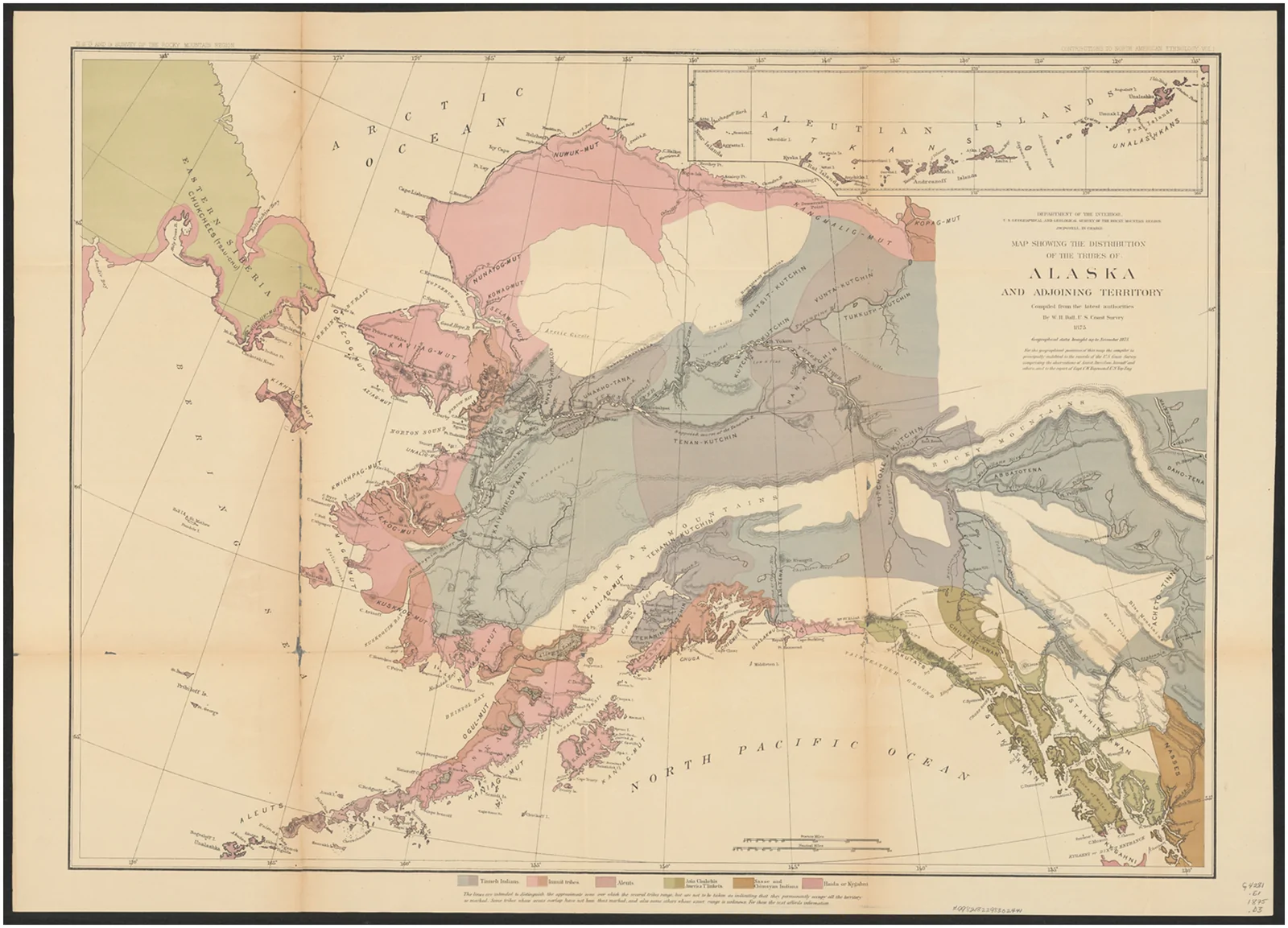
Map showing the distribution of the tribes of Alaska and adjoining territory. William Healey Dall, 1875. Image Courtesy of Northwestern University, Map and Atlas Collection.

One thousand years later, or so he reckoned, southern tribes forced the Continental Innuit back north and westward.^86^ Some spread out along the Northwest Coast, others into the Aleutian Islands. There they met with the Low Innuit, little-altered over the intervening millennium.^87^ It was this Aleutian Islands reunion that produced the surge toward civilization, and over the next two millennia the groups recombined to form the Aleut of the “historic period,” that is to say, the eighteenth century when Beringia and its inhabitants became the subject of written record under commission of the Russian Empire (Figure 5).^88^ Culturally speaking, Dall equated his “Orárian” contemporaries, Walker among them, with Europe’s cave-dwellers, thereby fixing a cornerstone in the long history of linking and inscribing biology, culture, and Beringia within a Euro-American-defined biogeographic hierarchy.^89^ This was a multifarious and divisive schema, asserting a geoenvironmentally determined hierarchy of cultural merit, and ultimately aptitude, of and within American indigeneity.

These excavations were the first of their kind in the region and Dall’s analyses, likewise distributed by the Smithsonian, were heralded as the first systematic and empirically founded history of humankind in Beringia. His three-part chronology, based on a combination of stratigraphic analysis and a unilinear artifact progression that conflated technology and subsistence strategies, fell well within the established norms (Figure 4). His environmental explanation for that cultural development and differentiation, however, signals something of an epistemic shift that was beggining to take place in studies of human culture, in that his methodological approach was all but indistinguishable from that used in his work on the biotic development and differentiation of Beringia’s molluscan beings. There is little reason to believe that this jump, from the naturalist’s protobiology to anthropology, was unique to Dall. His natural history was fairly typical for its time, so too was the assembly and analysis of collections that ranged from animals and plants to human artifacts.^90^ It is this potential ubiquity that renders a deeper understanding of how subjective experience factored into nineteenth-century environmentally deterministic science so desirable.

## Conclusion

Following the use of mollusks as evidence leads us across disciplines, methods, and theories, elucidating critical entanglements in the histories of natural and human science. It also presents a means of excavating the potential for the mode of subjectivity that is sensation, accumulated in the everyday work of science-making in environs beyond the ken of Euro-American knowledge systems and skill sets, to figure alongside trained methods and theories into the formation of scientific knowledge to shape and substantiate scientific discourses about unfamiliar environs within a quickly converging global hierarchy of place and being.

Dall’s introduction to Beringia emerged from a venture in which commercial, political, and scientific interests were tightly enmeshed. The venture extended access to Beringia at an early point in its history as an object of U.S. interests, placing Dall in an opportune position to assume expertise about that environment and the beings who lived there. Dall took mollusks and their remains as evidentiary points of departure for deciphering the status of Beringia and its inhabitants within an environmentally rationalized hierarchy that conflated other-than-human and human, biotic and cultural development. But how did a fledgling malacologist’s environmentally based forays into classification and development bear on the longer and broader history of science?

Dall was still in the field in late July of 1867 when he received word that the United States had purchased the territory now called Alaska; his diary entry for August 1^st^ notes, “Today Russian America becomes American soil . . . boys all drunk.”^91^ Upon his return to the East Coast, Dall became a fixture at the Smithsonian Institution, and was ultimately named Honorary Curator of Mollusks. In 1870 he published *Alaska and its Resources*, a several hundred-page study of the region’s natural, environmental, human, and above all economic value for government, commercial, and private use. This book cemented his reputation as an expert on Alaskan nature, culture, and history.^92^

From his position at the Smithsonian Institution, Dall went on to exercise considerable influence over late nineteenth and early twentieth-century classificatory practice and museum exhibition in the United States and beyond – and not just zoology, but also ethnology.^93^ Not everyone subscribed to his methods or theories, but his published reports fueled scientific inquiry into the region, his work became a cornerstone in international discourses on the origin of the Inuit, and a stop in Washington D.C. to view his collections and obtain his counsel became a rite of passage for subsequent researchers en route to Alaska. That is to say, much as the mollusks provided Dall with a point of entry for theorizing about how this environment had impacted development, the collections he amassed and the publications based on them went on to initiate generations of subsequent researchers to those environs.^94^

This brings us back to the question with which this contribution began: What was it that allowed Dall to shift his methods and theories with such apparent ease between mollusk-based research into the histories of biological and cultural development? Because it was never purely mollusks as such that formed the focus of his investigations. In Dall’s research in and along the Bering Strait, mollusks were interpreted as empirical evidence of a long-nurtured conviction: this unfamiliar environment was abnormal, and had exercised adverse effects on the development of the living- and once-living beings that inhabited it. This carried social, economic, and political implications that reverberated far beyond the immediate geographic borders and the humble lives of mollusks, supporting colonial interests by enrolling these places as inferior within a biogeographic hierarchy that descended from Euro-North American norms.

Dall presents the Beringian climate, and the cold in particular, as an impediment to progress of a very specific kind, namely socioeconomic, which he correlated with the capitalization of natural resources and adoption of Euro-American cultural norms.^95^ Dall’s was a practice deeply embedded in centuries-old Euro-American discourses about what constituted normal climates and environments on the one hand, and moral, intellectual, technological, economic, and social development on the other.

Confident as he was in the westward march of science and empire, Dall’s own expertise and the prerogative of science were still far from established in this unfamiliar space. It was not just mollusks as such that formed the crux of Dall’s investigations, but more essentially the question of how to value lives indigenous to this potentially lucrative environment. As for the environment itself, there was little that could be done to remedy heavy clouds, rain, fog, or severe gales, but he was optimistic about the ice. Much of the space “now considered valueless” could be transformed into arable farmland: large-scale removal of the ubiquitous moss ground cover, followed by draining and deep plowing, could in time eliminate the ice from the region’s soil entirely.^96^ He envisioned an Alaska that, 250 years hence, served U.S. citizens as a “New England of the Northwest,” but with far more lucrative natural resources.^97^

## Footnotes

1. Consider for instance the arboreal candy cane snail of Hispaniola, and on molluscan inhabitants of the ocean depths: see, e.g., Carolina E. González et al., “Revealing Zooplankton Diversity in the Midnight Zone,” *Frontiers in Marine Science* 10 (2023): 1252535, https://doi.org/10.3389/fmars.2023.1252535.

2. E.g., Jen Rose Smith, *Ice Geographies, The Colonial Politics of Race & Indigeneity in the Arctic* (Durham, NC: Duke University Press, 2025), pp.11–3, 85–6, 90–5, 134–5, 143–4; and on this environment as moreover “hostile” and “dangerous” see Andrew Stuhl, *Unfreezing the Arctic: Science, Colonialism, and the Transformation of Inuit Lands* (Chicago, IL: University of Chicago Press, 2018), pp.15–60.

3. Sadiah Qureshi, “Peopling Natural History,” in Helen Ann Curry et al. (eds.), *Worlds of Natural History* (Cambridge: Cambridge University Press, 2018), pp.363–78; Smith, *Ice Geographies* (note 2); Nicole Starosielski, *Media Hot and Cold* (Durham, NC: Duke University Press, 2022); and Hi’ilei Julia Kawehipuaakahaopulani Hobart, *Cooling the Tropics. Ice, Indigeneity, and Hawaiian Refreshment* (Durham, NC: Duke University Press, 2022).

4. Lorraine Daston, “The Moral Economy of Science,” *Osiris* 10 (1995): 2–24. Much has been written on the mid-nineteenth-century emergence of the ideal of scientific objectivity see esp. Id., “Fear and Loathing in the Imagination of Science,” *DÆDALUS* 127 (1998): 73–95; and Lorraine Daston and Peter Galison, *Objectivity* (New York, NY: Zone Books, 2009).

5. Steven Shapin, “The Sciences of Subjectivity,” *Social Studies of Science* 42 (2012): 170–84, esp. p.171, 176; and Shapin citing Richard Rorty, *Objectivity, Relativism, and Truth: Philosophical Papers* (Cambridge: Cambridge University Press, 1990), pp.35–45.

6. Peter R. Martin, “The ‘Deer-Men’ and the ‘Bowhead-Men’: The Colonial Co-optation of Arctic Indigenous Knowledge within the ‘Origins of the Inuit’ Debates,” *Transactions of the Institute of British Geographers* 49 (1)(2024): e12615, 2, https://doi.org/10.1111/tran.12615.

7. Oceans are a subject of growing interest in the history of science, see, e.g., Helen Rozwadowski’s *Vast Expanses. A History of the Oceans* (London: Reaktion Books, 2018); Anthony Adler’s *Neptune’s Laboratory: Fantasy, Fear and Science at Sea* (Cambridge, MA: Harvard University Press, 2019); and Samantha Muka’s *Oceans under Glass: Tank Craft and the Sciences of the Sea* (Chicago, IL: Chicago University Press, 2023), yet little has been written on the specificity of research conditions presented by straits. For an authoritative environmental history of the mid-nineteenth to late twentieth-century Bering Strait see Bathsheba Demuth, *Floating Coast. An Environmental History of the Bering Strait* (New York, NY: W.W. Norton, 2019).

8. Consider, for example, the U.S. Department of State’s Extended Continental Shelf project, https://www.state.gov/the-us-ecs/; Yereth Rosen, “As U.S. Prepares to Claim New Ocean Territory off Arctic Alaska and in Bering Sea, There’s a Catch,” *Alaska Beacon*, 28 December 2023, https://www.adn.com/politics/2023/12/28/as-us-prepares-to-claim-new-ocean-territory-off-arctic-alaska-and-in-bering-sea-theres-a-catch/; and Id., “Russian Objection to U.S. Territorial Claims off Alaska Complicates Maritime Relationship,” *Alaska Beacon*, 6 April 2024, https://alaskabeacon.com/2024/04/06/russian-objection-to-u-s-territorial-claims-off-alaska-complicates-maritime-relationship/ (all 23 July 2025).

9. For an overview of the circumstances surrounding Russian colonization of North America see, e.g., Jon Katchen and Nicholas Ostrovsky, “Strangers in Their Own Land: A Survey of the Status of the Alaska Native People from the Russian Occupation Through the Turn of the Twentieth Century,” *Alaska Law Review* 39 (2022): 1–47, esp. pp.9–10, 16–26.

10. Robert Kindler, “American Russia: Fur Seals, Empire and Conflict in the Northern Pacific after 1867,” *Ab Imperio* 1 (2022): 166–90, esp. 171–2; and Katchen and Ostrovsky, “Strangers,” p.27 (note 9).

11. William H. Dall, *Alaska and its Resources* (Boston, MA: Lee and Shepard, 1870), pp.481–505.

12. On the arc of Agassiz’s U.S. reception see, e.g., Mary P. Winsor, *Reading the Shape of Nature. Comparative Zoology at the Agassiz Museum* (Chicago, IL: University of Chicago Press, 1991), p.3, 81; and Louis Agassiz, *Methods of Study in Natural History* (Boston, MA: Ticknor and Fields, 1863), p.41.

13. W. P. Woodring, *William Healey Dall 1845–1927. A Biographical Memoir* (Washington, DC: National Academy of Sciences, 1953); and on training under Agassiz see: Winsor, *Shape of Nature*, pp.3–4, 9, 16 (note 12).

14. On Wyman’s perspective on evolution, and shift from teaching development as the result of consecutive acts of creation to evolution precisely during the time of Dall’s tutelage see: Hunter A. Dupree, “Jeffries Wyman’s Views on Evolution,” *Isis* 44 (1953): 243–6; and Toby Appel, “Jeffries Wyman, Philosophical Anatomy, and the Scientific Reception of Darwin in America,” *Journal of the History of Biology* 21 (1988): 69–94, 85, 77, 91; and on Wyman’s practice more generally A. S. Packard, *Memoir of Jeffries Wyman 1814-1874* (Washington, DC: Judd & Detweiler, 1878).

15. “Memoranda” February 1, final pages, Dall diary 1865, Record Unit 007073, Box 20, Smithsonian Institution Archive (henceforth DD 1865).

16. Dall frequently repeats this sentiment, here in reference to William Abbot Nason, January 27, DD 1865.

17. On circumstances preceding the expedition see, e.g., Rosemary Neering, *Continental Dash, the Russian-American Telegraph* (Ganges, BC: Horsdal & Schubart, 1989), pp.18–25.

18. On expedition financing and infrastructure see Stewart Andrew Robb, “The Collins’ Overland or Russian Extension Telegraph Project: A Pioneer Attempt to Establish Telegraphic Communication between North America and Europe,” (MA thesis, Simon Fraser University, 1968), p.10, 17. See also Debra Lindsay, *Science in the Subarctic. Trappers, Traders, and the Smithsonian Institution* (Washington, DC: Smithsonian Institution Press, 1993), p.106; Hunter Miller, “Russian Opinion on the Cessation of Alaska,” *American Historical Review* 48 (1943): 521–31, 521–2. For another first-hand account of expedition organization see Frederick Whymper, *Travel and Adventure in the Territory of Alaska* (London: W. Clowes and Sons, 1868), p.70.

19. February 15–17, DD 1865.

20. Based on George Berkeley’s 1752 “On the Prospect of Planting Arts and Learning in America,” March 9; on Dall’s rank, responsibilities, and party see Scientific Corps W.U.I Cos. Ex., undated; and copy of Kennicott to Dall, September 8, all Ibid. (latter two among final pages). On Dall’s admiration of Kennicott see also Dall, *Alaska* (note 11), dedication; and Neering, *Continental Dash*, p.181 (note 17).

21. Two notable forerunners were Alexander von Middendorf’s and William Stimpson’s investigations on behalf of the Russian and U.S. governments, respectively. The latter will be discussed shortly, on the former see Maxim V. Vinarski, “The Great Empire’s Malacologist: Alexander von Middendorf’s Contribution to the Study of Molluscs,” *Ruthenics* 31 (2021): 177–96, esp. 188.

22. Mareike Vennen, *Das Aquarium. Praktiken, Techniken und Medien der Wissensproduktion (1840–1910)* (Göttingen, Germany: Wallstein, 2018), p.235, and on international transport of sea creatures more generally, pp.235–51. The shell of the creature pictured survives in damaged condition at the Smithsonian’s National Museum of Natural History (NMNH). On the production of this sketch see July 22–24; and on the preservation of living specimens July 16, all DD 1866 — May 25, 1867, RU 007073 S. 6 B. 20, SIA (DD 1866–7).

23. Agassiz, *Methods*, p.23 (note 12); Winsor, *Shape of Nature*, pp.4, 12–19 (note 12); Lane Cooper, *Louis Agassiz as a Teacher: Illustrative Extracts on His Method of Instruction* (Cornell: Cornell University Press, 1917), pp.10–13, 40–8.

24. On tins see May 6; on paper tubes see, among others, December 19; on boiling mollusk bodies to procure shells see January 16, 1866, all DD 1865.

25. July 20, final pages, Ibid.

26. See, e.g., William Healey Dall, “Catalogue of Shells of the Bering Strait and Adjacent Portions of the Arctic, with Descriptions of Three New Species,” *Proceedings of the California Academy of Sciences* 5 (1874): 246–53, note p.252 ; William Healey Dall, “On the Limpets; with Special Reference to the Species of the West Coast of America, and to a More Natural Classification of the Group,” *American Journal of Conchology* 6 (3)(1871): 227–82; William Healey Dall, “On the Extrusion of the Seminal Products in Limpets, with Remarks on the Phyllogeny [*sic*] of the Docoglossa,” *Scientific Results of the Exploration of Alaska* 1 (1876): 239–47, 235–43; and David R. Lindberg, “William Healey Dall: A Neo-Lamarckian View of Molluscan Evolution,” *The Veliger* 41 (3)(1998): 232.

27. Dall, “Extrusion of Seminal Products,” 244–5 (note 26); Lindberg, “Dall,” pp.235–6 (note 26).

28. Ibid., pp.232–3.

29. Lindberg furthermore diagnoses Dall’s later practice as rooted in Lamarck’s and Spencer’s progressive developmental models, Ibid., esp. pp.235–7.

30. On Stimpson and Agassiz see Ronald Scott Vasile, *William Stimpson and the Golden Age of American Natural History* (Dekalb, IL: Northern Illinois University Press, 2018), pp.65–8. As Dall told Smithsonian Assistant Secretary Spencer Fullerton Baird, he wished to do for mollusks of the North Pacific what Stimpson had done for those of the East Coast; Dall to Baird, December 16, DD 1865.

31. Vasile, *Stimpson*, p.68, 206 (note 30).

32. Wyman continues: “and that thus the order of Nature, as commonly manifest in her works, was maintained,” see Jeffries Wyman, “Monograph of the Aye-Aye (Chiromys Madagascariensis Cuvier); by Richard Owen,” *The American Journal of Science*, s. 2 v. 36 (1863): 294–99, esp. 299; and Appel, “Wyman,” 77 (note 14). For Agassiz on complication of structure see e.g., Agassiz, *Methods*, pp.41–71, 84–102 (note 12). For Dall’s perspective on development and nature’s plan see e.g., Dall, “Catalogue of Shells of the Bering Strait,” (note 26).

33. Lindberg, “Dall,” pp.231–7, quotes 237 (note 26).

34. Thanks to Ellen Strong, Smithsonian Institution National Museum of Natural History Chair of Invertebrate Zoology, Research Zoologist and Curator of Mollusks, for these images and for sharing her insights into the Dall collection and its history.

35. On the history of the concept of environment in Europe, France in particular, see Etienne Benson, *Surroundings. A History of Environments and Environmentalisms* (Chicago, IL: Chicago University Press, 2020), pp.29–41; with respect to marine life, Möbius, and experimentation see Vennen, *Aquarium*, pp.317–21 (note 22); and on the state of knowledge about the relationship between morphology, development, and experimentation Lynn K. Nyhart, *Biology Takes Form: Animal Morphology and the German Universities, 1800-1900* (Chicago, IL: Chicago University Press, 1995), p.3, 6–11.

36. Shapin, “Sciences of Subjectivity,” (note 5); and Shapin citing Rorty, *Objectivity, Relativism, and Truth*, pp.35–45 (note 5).

37. Shapin, “Sciences of Subjectivity,” 178 (note 5); see also Id., “The Tastes of Wine, Toward a Cultural History,” *Rivista di Estetica* 51 (2012): 49–94, esp. 64; and Id., “A Taste of Science: Making the Subjective Objective in the California wine world,” *Social Studies of Science* 46(2016): 436–60, esp. pp.450–2.

38. “We’re Bound to Go” was based on the hymn “Homeward Bound” attributed to one R.J. Bush, DD 1865.

39. For an exceptional study on trained judgment see, Anne Secord, “Specimens of Observation: Edward Hobson’s Musci Britannici,” in Joshua Nall, Liba Taub, and Frances Willmoth (eds.), *The Whipple Museum of the History of Science: Objects and Investigations* (Cambridge: Cambridge University Press, 2019), pp.101–18; and for broader overviews see Daston and Galison, *Objectivity*, pp.309–61 (note 4); and Lorraine Daston and Elizabeth Lunbeck, “Introduction: Observation Observed,” *Histories of Scientific Observation*, Id. (eds.) (Chicago, IL: Chicago University Press, 2010), pp.1–10.

40. Paraphrasing and quoting Smith, *Ice Geographies*, p.11 (note 2); see also Lisa Bloom, *Gender on Ice. American Ideologies of Polar Expeditions* (Minneapolis, MN: University of Minnesota Press, 1993).

41. Dall, *Alaska*, p.169, 242 (note 11).

42. For exact dates see Woodring, *Dall*, pp.95–7 (note 13).

43. January 25–26, DD 1865; and December 9, July 16, 1866, DD 1866–7. On adversity in comparatively mild temperatures see William Healy Dall, *Tribes of the Extreme Northwest* (Washington, DC: Government Printing Office, 1877), p.41. May, June, and part of July he found “delightful, – sunny, warm, and clear,” Dall, *Alaska*, p.436 (note 11).

44. Dall may have had access to meteorological records collected between 1842 and 1844, see Dall, *Alaska*, pp.539–42 (note 11). On the history of empire and climate science more generally see, e.g., Deborah R. Coen, *Climate in Motion. Science, Empire, and the Problem of Scale* (Chicago, IL: Chicago University Press, 2018); and Martin Mahony and Georgian Endfield, “Climate and Colonialism,” *WIREs Climate Change* 9 (2018): e510.

45. On distinguishing between fact, theory, and opinion see Dall, *Alaska*, pp.v–vi (note 11); on fact based on field observation as opposed to museum analysis see Dall to Baird, December 16, DD 1865.

46. On fearing the subversion of scientific fact see Daston, “Fear and Loathing,” 74 (note 4). On Kennicott’s praise of Dall see Kennicott to Caroline Healey Dall, July 11, 1865, RU 007073, Series 2 Box 2, SIA; and copy of Kennicott to Dall, October 8, DD 1865. There is an indication that Dall acknowledged the risk of subjectivity creeping into climate assessments, but considered himself immune, see, Dall, *Tribes*, p.54 (note 43).

47. June 14–15, DD 1865.

48. September 16, 18, 23, Ibid. The WUTE Engineer Corps was likewise ill-equipped. E. B. Libby, superintendent of a working division, protested that whomever outfitted the party “had no idea of the weather . . . Army coats were useless in Arctic weather; what was needed was fur clothing,” see Neering, *Continental Dash*, p.45, 192 (note 17).

49. August 21, September 23–28, DD 1865. Plover Bay is now known as Providence Bay, and elsewhere Dall notes Walker’s name as “Nokum.” Walker is likewise mentioned as a guide and interpreter, though this time as Chukchi and spelled “Nau-kum,” and in Whymper, *Travel and Adventure*, pp.88–91 (note 18). Cross-referencing these and other sources that mention villages inhabited at the time suggests that these people were delegates of the two local Yupik communities.

50. Scientific Corps W.U.I Cos. Ex., undated, final pages, DD 1865.

51. February 21, July 16, 1866, Dall diary, 1866-May 25, 1867, RU 007073 B. 20 SIA (henceforth DD 1866–7).

52. June 7, December 1, Ibid.

53. August 15–22, September 8, 12, Ibid.

54. Sept. 25–26, 30, Ibid.

55. Ibid.; William Healey Dall, “Explorations in Russian America,” *American Journal of Science* s2-45 (1868): 96–99; and clipping of published letter, Dall to J. Young Scammon, September 27, 1866, RU 007073 S. 6 B. 20, SIA, Western Union Telegraph Expedition Collection (online, last accessed September 2, 2025). On Dall’s determination to complete Kennicott’s commission see also Neering, *Continental Dash*, p.181, 207–6 (note 17). On Dall’s Yukon expedition team see December 31, DD 1866–7.

56. October 4–5, DD 1866–7.

57. Dall refers to the *Qasgiq* as “*casine*,” “as the Russians term it,” October 26–November 1, 1886, DD 1866–7; and Dall, *Alaska*, p.16 (note 11).

58. October 7, 12–17, 24–31, DD 1866–7.

59. On the “preposterous folly of issuing ordinary rations to men in an arctic, or nearly arctic climate,” see Dall, *Alaska*, p.66 (note 11); and Whymper, *Travel and Adventure*, p.61 (note 18).

60. October 27–November 6, 1866, DD 1866–7.

61. As he put it, “it is very stupid staying in an Indian house with nothing to do but eat drink, and sleep and the days only 4 hours long,” November 14–21; there was “nothing to do [and] life very stupid,” December 18–19, both Ibid.

62. November 6–21, Ibid.; and Dall, *Alaska*, p.195 (note 11). USNM E5581-0, acquired in the final year of his expedition, establishes that he eventually learned of snow goggles.

63. December 18–19, 1866, Jan 12–14, March 17, 20, 1887, Ibid.

64. December 14, Dall diary Dec 1 1867 — Aug 4, 1868 RU 007073 S. 6 B. 20, SIA (henceforth DD 1867–8).

65. December 15, Ibid.

66. Dall, *Alaska*, p.102 (note 11); Woodring, *Dall*, pp.97–8 (note 13).

67. Dall’s diaries abound with mentions of God, church, sermons, and the lack of services in the field; see among others January 15 and February 5. There was intermittent access to hot water baths at Russian forts, and he, by and large, foreswore Indigenous bathhouses; on baths, see, e.g., November 28, all DD 1865; and Dall, *Alaska*, pp.19–20 (note 11). On the distress of long hair see September 21, 1866, DD 1866–7.

68. Howard M. Feder et al., “Mollusks in the Northeastern Chukchi Sea,” *Arctic* 47 (1994): 145–63. On “temperate-normativity” see Smith, *Ice Geographies* (note 2).

69. Dall, “Extrusion of Seminal Products,” 244–5 (note 26); and Lindberg, “Dall,” 227–38, 235–6 (note 26). For other prominent naturalists of the time who held comparable views on the relation of climate and evolution see, e.g., Philip Lutley Sclater, “On the General Geographical Distribution of the Members of the class Aves,” *Journal of the Proceedings of the Linnean Society: Zoology* 2 (1858): 130–45; and Alfred Russel Wallace, *The Geographical Distribution of Animals* (New York, NY: Harper & Brothers, 1876).

70. These classifications never entered common usage and may have been based on place names, see Dall, *Alaska*, pp.93–6, 108, 202 (note 11).

71. Ibid., 103.

72. Dall refers to this group as the Kygáni, Ibid., p.411.

73. Adolph Morlot, “General Views on Archaeology,” *Annual Report of the Board of Regents of the Smithsonian Institution for 1860* (Washington, DC: George W. Bowman, 1861), pp.284–343. For international discourses on archaeological theories and methods of the time see, e.g., Bruce G. Trigger, *A History of Archaeological Thought* (Cambridge: Cambridge University Press, 1996), p.14. For an overview of the history of speculation about the origin of Indigenous Americans, Christian Jürgensen Thomsen’s development of systematic relative and chronological stratigraphic dating of human artifacts, and the shell heap investigations of Johann Georg Forchhammer, Japetus Steenstrup, and J.J.A. Worsaae that inspired Morlot see Ibid. pp.114–7, 121–38.

74. Appel, “Wyman,” 88 (note 14). Conceptions of the age of the Americas themselves were also at this time changing, on this and the idea that “the true native Americans were not the Indians but these far more ‘ancient Americans’ as one paleontologist called the fossilized mammals of the West”; see Caroline Winterer, *How the New World Became Old* (Princeton, NJ: Princeton University Press, 2024), pp.44–60, and 121–44, esp. 122

75. Asa R. Randall, “How Jeffries Wyman put Florida and Shell Mounds on the Map (1860–1875),” *Bulletin of the History of Archaeology* 25 (2015): 1–12, 4; Katie Kirakosian, “Mapping the Social Worlds of Shell Midden Archaeology in Massachusetts,” *Bulletin of the History of Archaeology* 25 (2015): 1–11, 3.

76. Early investigators debated whether U.S.-based shell sites were in the first order geologic, with subsequent human-made additions, see, e.g., Daniel Garrison Brinton, “Artificial Shell Deposits of the United States,” *Annual report of the Smithsonian Institution for 1866* (Washington, DC: Government Printing Office, 1867), pp.356–8; Jeffries Wyman, *Fresh-Water Shell Mounds of the St. John’s River* (Salem, MA: Peabody Academy of Science, 1875), pp.45–60, 86–7; and Randall, “Wyman,” 3 (note 75).

77. While Wyman stressed the fallibility of dating methods, his preferred method was counting the rings of trees newly felled from atop shell heaps, see Wyman, *Fresh-Water Shell Mounds*, p.53 (note 76); Appel, “Wyman,” p.88 (note 14); Randall, “Wyman,” p.5 (note 75); Packard, *Wyman*, pp.110–2 (note 14). On Thomsen see, e.g., Trigger, *Archaeological Thought*, pp.121–7 (note 73).

78. Dall, *Tribes*, p.41, 47 (note 43).

79. “Ora” in reference to what he determined as their coastal distribution, see Dall, *Alaska*, p.373 (note 11).

80. Ibid., pp.373–432, esp. 373–4, and 377; and Dall, *Tribes*, p.9, 95 (note 43).

81. Dall, *Tribes*, pp.49–56 (note 43).

82. Ibid., 55.

83. Ibid., 60–62.

84. Ibid., 98.

85. Ibid., 48–9, 60–2, 99.

86. Dall, *Alaska*, pp.373–432, esp. 373–4, and 377 (note 11); and Dall, *Tribes*, p.9, 95 (note 43).

87. On the route of this second wave of migration see Ibid.; and 53, 73 for his calculations of the age of the Littoral Period layer.

88. Dall, Ibid., p.49.

89. Dall, *Alaska*, pp.237–8 (note 11). Wyman, for instance, was developing a method to determine cranial capacity (equated with intelligence), and diagnosed a correlation that differentiated Tungusic, Siberian Chukchi, and North American Inuit from all other Indigenous Americans. He also calculated the Chukchi foramen magnum index as “very nearly the same as in the white races,” and that of North American Indians as the lowest, which he interpreted as evidence that this population made “the nearest approach” to apes. See Jeffries Wyman, *Observations on Crania* (Boston, MA: A. A. Kingman, 1868), pp.11–4, 24. For Dall citing these studies see Dall, *Tribes*, pp.63–8 (note 43).

90. Among prominent contemporaries consider, for instance, Darwin, Alfred Russel Wallace, and Otto Finsch.

91. August 1, DD Jul 14–Nov 30, 1867 (DD 1867). Russia sold the province to the United States for $7,000,000 plus $200,000 to buy out the land claims of fur and ice monopolies maintained by the Russian American Company and American Russian Commercial Company; see e.g., Adolf Bastian, “Asiatisch-amerikanische Polargegend,” *Zeitschrift der Gesellschaft für Erdkunde zu Berlin* 2 (1867): 423–38, 436–7; Kindler, “American Russia, ” (note 10); and E. L. Keithahn, “Alaska Ice, Inc.,” *The Pacific Northwest Quarterly* 36 (1945): 121–31.

92. His mark on that landscape was memorialized in 1879 with the naming of Dall Island, just off the southeast coast of Alaska. Seven years later he published *Tribes*, which includes a section entitled “Remarks on the Origin of the Innuit,” pp.93–106 (note 43).

93. Over the course of his career Dall named more than 35 genera and subgenera, and nearly 4,000 species and subspecies, and his works continue to be cited today, see Woodring, *Dall*, p.103 (note 13).

94. Among others, Adolf Bastian and Franz Boas, who went on to become luminaries of German and U.S. anthropology, respectively, and direct vast collection and expedition efforts of their own in Beringia.

95. Dall, *Alaska*, pp.433–4 (note 11).

96. Ibid., pp.434–5.

97. Ibid., p.242.

